# The Management of Hematopoietic Stem Cell Transplant in People with HIV

**DOI:** 10.3390/v16101560

**Published:** 2024-09-30

**Authors:** Jana K. Dickter, Courtney Moc Willeford

**Affiliations:** 1 Division of Infectious Diseases, Department of Medicine, City of Hope National Medical Center, Duarte, CA 91010, USA; 2 Department of Pharmacy Services, City of Hope National Medical Center, Duarte, CA 91010, USA

**Keywords:** HIV, hematopoietic stem cell transplant, hematologic malignancy, opportunistic infection

## Abstract

Hematopoietic stem cell transplant (HSCT) is now recognized as a standard treatment option for people with HIV (PWH) who develop high-risk hematologic malignancies. However, the involved polypharmacy can lead to complications from drug interactions and toxicities, affecting the efficacy and safety of chemotherapy and antiretroviral therapy (ART). Managing these patients requires a personalized approach, including the careful selection of ART based on previous therapies and potential interactions, alongside risk assessment for infections. This discussion will address the history of HSCT in PWH and management considerations for this group.

## 1. Introduction

People with HIV (PWH) are at increased risk of developing hematologic malignancies, and hematopoietic stem cell transplantation (HSCT) is considered a standard therapy for certain high-risk cases. Studies have shown that PWH who undergo autologous HSCT (autoHSCT) and/or allogeneic HSCT (alloHSCT) achieve clinical outcomes, including survival rates, comparable to those of the general population [1,2,3,4]. However, the polypharmacy required during HSCT can result in complications due to drug interactions and overlapping toxicities, potentially affecting the efficacy and safety of chemotherapy, antiretroviral therapy (ART), and other immunosuppressive and/or chemoprophylactic treatments.

Managing these patients requires a personalized, multi-faceted approach, which includes evaluating the most appropriate ART regimen based on prior therapies, history of antiretroviral resistance, allergies or intolerances, and potential drug–drug interactions. Additionally, it is crucial to assess patients’ infection risk and provide appropriate screening, treatment, and preventative measures.

This discussion will include the history of HSCT in PWH and the unique evaluation and management considerations for this population.

## 2. Epidemiology

Before the availability of ART, most cancers in PWH were linked to severe immunosuppression. Early AIDS-defining conditions included diffuse large B-cell lymphoma (DLBCL), AIDS-related primary central nervous system lymphoma (PCNSL), Burkitt’s lymphoma (BL), primary effusion lymphoma (PEL), plasmablastic lymphoma of the oral cavity type (PBL). Other lymphoproliferative disorders strongly associated with HIV infection include Kaposi’s sarcoma-associated herpesvirus-associated multicentric Castleman disease (MCD) and Hodgkin’s lymphoma (HL) [5,6]. The relative risk of developing non-Hodgkin lymphoma (NHL) was estimated to be 60–200 times greater than that of the general population [7]. While autoHSCT in PWH before the availability of ART was complicated by multiple opportunistic infections post-HSCT, it was noted that stem cell mobilization and engraftment were feasible with HIV-1 replication [8]. 

With the success of ART, PWH are now living longer with higher CD4^+^ cell counts and fewer opportunistic infections [5,9]. This has led to a decrease in the incidence of AIDS-defining cancers (ADC) and an increase in non-AIDS-defining cancers (NADC) [10,11,12,13]. However, ART does not fully restore health, and PWH are at an increased risk of developing age-associated complications, including cancer. Factors contributing to this elevated risk include chronic immunosuppression, immune dysregulation, co-infection with oncogenic infections, premature aging, and a higher prevalence of carcinogen exposure (tobacco, alcohol) [12,14,15,16]. HIV can contribute to cancer risk by directly activating cellular oncogenes or proto-oncogenes, inhibiting tumor suppressor genes, and disrupting normal cell cycle regulation [15,17]. 

The incidence of hematologic malignancies in PWH varies based on age, CD4^+^ cell count, HIV control, viral co-infections, and chronic inflammation. In Europe and the United States, the incidence of NHL has decreased by half since the advent of ART as demonstrated by the reduced cases of PCNSL and the immunoblastic histologic subtype of DLBCL seen. However, the incidence of HIV-associated BL and HL has increased and is associated with moderate immunosuppression and poorer outcomes. In recent years, aggressive types of lymphomas, such as PBL and PEL, have become more prevalent [5]. HSCT remains a potential cure for patients with poor prognosis hematologic malignancies, and since the availability of ART, studies show that both autoHSCT and alloHSCT are safe for PWH [4]. 

To date, there are multiple studies that have demonstrated that the results of autoHSCT are comparable to the general population, with no long-term effects on CD4+ counts or HIV control [18,19,20,21,22,23]. The largest prospective multi-institutional trial reported by the Bone Marrow Transplant Clinical Trials Network (BMT CTN) and AIDS Malignancy Consortium found that autoHSCT was safe and effective in PWH, with no difference in one-year overall survival rates. However, a small study from Japan evaluating the impact of autoHSCT in PWH treated for NHL and multiple myeloma found an increased risk of overall mortality and a higher incidence of relapse in PWH [24]. Studies have also shown an increased risk of infectious complications among PWH undergoing autoHSCT, including bacterial infections during the pre-engraftment period, viral infections in the post-engraftment period, and cytomegalovirus (CMV) reactivation [25]. 

For alloHSCT, the largest prospective trial found that relapse of the underlying malignancy was the most common cause of treatment failure, with no increased relapse risk associated with HIV. CD4^+^ cell counts returned to baseline, and there were no adverse effects on humoral or cellular immune function. This study supported the safety and feasibility of alloHSCT for PWH [3]. However, PWH undergoing alloHSCT may have a higher risk of opportunistic infections, with one trial demonstrating an increased risk of nontuberculous mycobacterial and CMV infection compared to HIV-negative patients [4]. With new prophylactic therapies available, particularly for CMV prophylaxis, it may be possible to minimize some of these risks. 

In conclusion, while the landscape of hematologic malignancies in people with HIV (PWH) has evolved significantly with the advent of ART, challenges remain in managing these complex conditions. Continued research and advancements in therapeutic strategies, including targeted prophylaxis for infections, offer hope for improving outcomes and reducing complications in PWH undergoing HSCT.

## 3. Optimizing ART for PWH Undergoing HSCT

Maintaining a fully active ART regimen for PWH is standard of care during HSCT, as data shows increased survival rates in PWH who remain on ART, and interruption of ART is associated with higher mortality rates [26,27,28,29,30]. However, managing ART during HSCT presents challenges due to frequent drug interactions between lymphodepletion and immunosuppression medications and HIV medications due to inhibition of and/or metabolism by the cytochrome P450 system (CYP450) or P-glycoprotein (P-gp). Furthermore, patients undergoing HSCT receive supplemental agents such as steroids, antiemetics, acid suppressants, antibiotics, and allopurinol. ART regimens may need adjustment to avoid significant drug interactions and ensure optimal treatment outcomes for hematologic cancer. It is crucial to also consider interactions with these supplemental medications, underscoring the importance of consulting with an HIV specialist in such cases.

**Figure 1:**
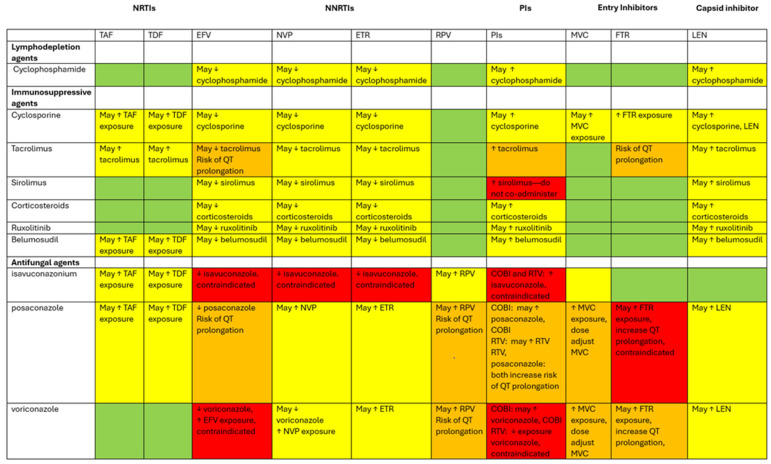
Diagram of drug interactions between ART and medications used during HCT. This diagram categorizes various agents involved for PWH undergoing HCT. The rows represent lymphodepleting agents (cyclophosphamide), immunosuppressive agents (cyclosporine, tacrolimus, sirolimus, corticosteroids, ruxolitinib, belumosudil), and antifungal agents (isavuconazonium, posaconazole, and voriconazole). The columns feature ART medications that are known to interact with the agents. The color coding used denotes the extent of the drug interactions. Green boxes: no drug interactions identified. Yellow boxes: potential for drug interactions exists; use these agents together with caution and close monitoring. Orange boxes: significant drug interactions; dose adjustments may be necessary, and/or there is a risk of QT prolongation. Red: contraindicated combination; avoid using these medications together. The ↑ symbol indicates an increase in drug concentrations, while the ↓ symbol signifies a decrease.

Newer ART options pose fewer risks of interaction with regimens commonly used in HSCT. Below are considerations for managing drug interactions with various classes of ART and supplementary medications. As many HIV medications are combination pills, assessing the composition of a patient’s regimen is necessary. Special attention must be given to potential side effects and drug interactions when initiating or maintaining ART, especially during HSCT. Drug interactions are further summarized in Table 1 and Figure 1.

**Nucleoside/nucleotide reverse transcriptase inhibitors (NRTIs):** NRTIs have minimal interactions as they are not metabolized by the CYP450 system. However, caution is advised with drugs that may worsen side effects during HSCT. Hepatotoxicity is a potential side effect of all NRTIs. Additionally, individuals taking tenofovir alafenamide (TAF) or tenofovir disoproxil fumarate (TDF), which are excreted renally and may interact with drugs that affect renal function or compete for tubular secretion, require monitoring of renal function [31]. TDF/TAF are also substrates of P-gp and breast cancer resistance protein (BCRP), so medications that affect P-gp and BCRP metabolism may alter TDF/TAF absorption [31].

Additionally, PWH who are co-infected with hepatitis B (HBV) should be treated with TDF or TAF plus lamivudine (3TC) or emtricitabine (FTC), as immunosuppressive or cytotoxic chemotherapy can increase the risk of HBV reactivation or disease [32]. Entecavir is FDA-approved for hepatitis B only but exhibits activity against HIV and can select for mutations conferring resistance to lamivudine and emtricitabine. If entecavir is administered to PWH, they must concurrently receive fully suppressive ART [33]. 

**Non-nucleoside reverse transcriptase inhibitors (NNRTIs):** All NNRTIs have the potential for hepatotoxicity and should be closely monitored when used during HSCT. They pose a higher risk of drug–drug interactions compared to NRTIs due to their extensive metabolism by, or inhibition of, the CYP450 system. First-generation NNTIs (efavirenz, nevirapine, and delavirdine) have a low barrier to resistance, while second-generation NNRTIs (etravirine, rilpivirine, and doravirine) are better tolerated and effective against first-generation NNRTI resistance. NNRTIs have prolonged half-lives, so discontinuation may lead to a period of functional monotherapy, increasing the risk of resistance [34]. Etravirine, rilpivirine, and doravirine are primarily metabolized by cytochrome P450 3A4 (CYP3A4). Additionally, etravirine is an inhibitor of P-gp [34]. Patients on NNRTIs during HSCT should be closely monitored, and adjustments to medication regimens may be necessary to avoid interactions. 

**Protease inhibitors (PIs):** Regimens containing PIs with pharmacokinetic boosters (e.g., ritonavir and cobicistat) pose substantial drug–drug interaction risks due to their inhibition of CYP3A4. If a PI and pharmacokinetic booster are administered with drugs metabolized by CYP3A4 isoenzymes, the coadministered drug plasma concentrations (e.g., tacrolimus) may increase, thereby increasing the risk of toxicity. Conversely, if a PI and pharmacokinetic booster are administered with drugs that are prodrugs, which are metabolized by CYP3A4, this can lead to increased levels of the prodrug but decreased levels of the active drug, thereby reducing efficacy [34,35]. 

Monitoring for increased drug toxicity and adjusting doses or replacing ART agents is crucial. Graft-versus-host disease (GVHD) prophylaxis medications also interact with boosted PIs, as highlighted by a case report where tacrolimus overdose occurred despite dose adjustment, emphasizing the need for careful management of drug interactions [36].

**Integrase strand transfer inhibitors (INSTI)**: INSTIs include elvitegravir (EVG), raltegravir (RAL), cabotegravir (CAB), dolutegravir (DTG), and bictegravir (BIC). They are well tolerated and have few drug interactions, making them attractive alternatives to PIs and NNRTIs. Studies show that ART regimens with INSTIs are more efficacious and have lower virologic failure rates compared to those with PIs. INSTIs also have a high barrier to resistance and are effective for patients with pre-existing resistance mutations [37,38,39,40]. Regimens like BIC/FTC/TAF, DTG+FTC/TAF, or DTG+FTC/TDF are used frequently during HSCT and are also effective for co-treating HBV. 

EVG, RAL, DTG, and BIC are substrates of UDP glucuronosyltransferase 1-1 (UGT1-A1). With minor contributions, EVG and BIC are metabolized by CYP3A. BIC is an inhibitor of Organic Cation Transporter 2 (OCT2) and Multidrug and Toxic compound Extrusion 1 (MATE1). Drugs that strongly induce UGT1-A1 and CYP34 (for EVG and BIC) can decrease the plasma concentrations of EVG, RAL, DTG, and BIC, leading to the loss of therapeutic effect and the development of resistance. Coadministration of BIC and drugs that are substrates of OCT2 and MATE1 may result in an increase in coadministered drug plasma concentrations. CAB is a substrate of P-gp and BCRP in vitro; however, due to its high permeability, there are no effects on CAB absorption expected with coadministration of P-gp or BCRP inhibitors [34].

**Entry Inhibitors:** Entry inhibitors include fusion inhibitors (enfuvirtide), CCR5 antagonists (maraviroc), attachment inhibitors (fostemsavir), and post-attachment inhibitors (ibalizumab-uiyk). Both enfuvirtide and ibalizizumab-uiky have no known drug–drug interactions and are administered subcutaneously and intravenously, respectively [34], bypassing gut absorption. This is particularly useful for patients who have post-HSCT sequalae such as gastrointestinal GVHD [41,42]. Additionally, these drugs may also be useful to overcome drug–drug interactions with HSCT lymphodepletion regimens. One report described the use of ibalizumab-uiyk during chemotherapy as an alternative to their PI regimen to avoid drug interactions [43]. 

In contrast, maraviroc and fostemsavir are CYP3A and P-gp substrates and are given orally. Additionally, fostemsavir is also a BCRP substrate, and coadministration of fostemsavir with drugs that are strong CYP3A inducers may result in decreased plasma concentrations of fostemsavir. However, the coadministration of fostemsavir with drugs that are moderate CYP3A inducers, P-gp, or BCRP inhibitors is not likely to have a clinically relevant effect on the plasma concentrations of fostemsavir. Additionally, fostemsavir is an inhibitor of Organic Anion Transporting Polypeptide 1B1 (OATP1B1), Organic Anion Transporting Polypeptide 1B3 (OATP1B3), and BCRP. The use of these medications requires careful monitoring to avoid drug–drug interactions [34,44,45]. 

**Capsid Inhibitors:** Lenacapavir is used for PWH who are heavily treatment-experienced with multidrug resistant HIV-1 infection. It is a substrate of P-gp, UGT1-A1, and CYP3A and a moderate inhibitor of CYP3A, P-gp, and BCRP. Drugs that are strong or moderate inducers of CYP3A may significantly decrease plasma concentrations of lenacapavir, leading to loss of therapeutic effect and the development of resistance [46]. Combined P-gp, UGT1A1, and strong CYP3A inhibitors may significantly increase plasma concentrations of lenacapavir; thus, these inhibitors are not recommended [34].

**Antifungal Agents:** Antifungal agents used for treatment or prophylaxis can significantly interact with ART. Isavuconazonium is contraindicated with boosted PIs due to its interaction with strong CYP3A4 inhibitors [47]. Posaconazole interacts with CYP3A4 substrates, including PIs and efavirenz, potentially affecting efficacy [48]. Voriconazole’s effectiveness is reduced when used with efavirenz and significantly reduced with ritonavir; thus, it is contraindicated with ritonavir [49]. Fluconazole has a weak interaction with the CYP450 system and is generally safe to use. PWH taking tenofovir and amphotericin B should be monitored for renal dysfunction due to potential nephrotoxicity [50]. 

**Renal Monitoring:** Renal function is closely monitored during HSCT. Some drugs, such as cobicistat, dolutegravir, and rilpivirine, may cause an asymptomatic rise in serum creatinine by inhibiting tubular creatinine secretion without affecting glomerular filtration rate [34]. 

**Alternative Administration:** Patients having trouble swallowing pills (due to conditions like mucositis) can use crushed or liquid forms of certain ART agents. Subcutaneous or intravenous medications may also be suitable alternatives, while intramuscular drugs are avoided due to thrombocytopenia during HSCT. 

**HSCT Lymphodepletion:** Prior to HSCT, lymphodepletion, also known as conditioning regimens, is administered, typically involving fludarabine and cyclophosphamide. Cyclophosphamide undergoes biotransformation by various CYP450 enzymes, with CYP3A4 contributing variably (5–25% of total enzyme activity) [51]. This variability means that NNRTIs such as delavirdine and PIs with pharmacokinetic boosters can increase the concentrations of cytotoxic metabolites, potentially raising the incidence of neutropenia, infections, and mucositis. A study comparing PI-based regimens plus cyclophosphamide-doxorubicin-etoposide (CDE) chemotherapy to PI-sparing regimen plus CDE found statistically lower neutrophil counts at Day +10 in patients receiving PI-based regimen compared to patients with PI-sparing regimens [52]. Furthermore, CYP3A4-inducing NNRTIs like nevirapine and etravirine may decrease the concentrations of cyclophosphamide exposure while increasing cytotoxic metabolites, necessitating close monitoring for myelotoxicity.

**Drug–drug Interactions with Post-HSCT Care:** After HSCT, immunosuppressants are utilized to prevent GVHD. These include calcineurin inhibitors (tacrolimus or cyclosporine), mTOR inhibitors (sirolimus), antimetabolites (mycophenolate), and/or corticosteroids such as prednisone. 

Calcineurin inhibitors like tacrolimus and cyclosporine and mTOR inhibitors such as sirolimus are substrates of CYP3A4 and P-gp [53,54,55,56]. PIs, pharmacokinetic boosters, and delavirdine are CYP3A4 inhibitors, potentially increasing exposure to tacrolimus/cyclosporine/sirolimus and raising the risk of immunosuppressant-associated toxicities like neurotoxicity (tacrolimus/cyclosporine) and QT prolongation (tacrolimus). Conversely, efavirenz, nevirapine, and etravirine (NNRTIs) are CYP3A4 inducers, which may reduce tacrolimus/cyclosporine/sirolimus concentrations, affecting immunosuppressive efficacy. Thus, therapeutic drug monitoring of tacrolimus/cyclosporine/sirolimus is essential to ensure it maintains at target concentrations. 

Additionally, cyclosporine is also an inhibitor of CYP3A4 and multiple drug efflux transporters such as P-pg [57]. Concomitant use with P-gp substrates such as fostemsavir, maraviroc, and TDF/TAF may increase exposure to these drugs. Tacrolimus and TDF are both nephrotoxic; their combined use may increase the risk of renal toxicity, necessitating renal function monitoring by clinicians. 

Mycophenolate, an antimetabolite, is a substrate of glucuronyl transferase, limiting drug–drug interactions [58]. However, studies suggest possible interactions, such as decreased nevirapine plasma concentrations, although clinical significance remains unclear, underscoring the need for vigilant post-HSCT care [59].

Among corticosteroids, prednisone is commonly used. It is converted to the active metabolite prednisolone by 11β-hydroxycorticosteroid dehydrogenase. Prednisolone metabolism involves CYP3A4, with an unclear extent of involvement [60]. CYP3A4 inhibitors like PIs, pharmacokinetic boosters, and delavirdine may elevate prednisolone concentrations, potentially increasing corticosteroid-related adverse effects. Conversely, CYP3A4 inducers like efavirenz, nevirapine, and etravirine may reduce prednisolone concentrations, affecting immunosuppressive efficacy. 

**GVHD Care:** Despite post-transplant care of immunosuppression, some patients still develop GVHD, necessitating additional immunosuppressive therapy such as ruxolitinib and belumosudil.

Ruxolitinib is metabolized by CYP3A4 and CYP2C9 enzymes [61]. Consequently, CYP3A4 inhibitors like PIs, pharmacokinetic boosters, and delavirdine may increase ruxolitinib concentrations, potentially leading to adverse events. Conversely, CYP34A inducers such as efavirenz, nevirapine, and etravirine may decrease ruxolitinib concentrations, compromising its immunosuppressive efficacy. Regular patient monitoring is crucial to assess GVHD progression and ensure treatment efficacy. 

Belumosudil is also metabolized by CYP3A4 [62]. In drug–drug interaction studies, coadministration with strong CYP3A4 inhibitor did not significantly affect belumosudil exposure [63]. Therefore, PIs, pharmacokinetic boosters, and delavirdine are unlikely to clinically impact belumosudil. Conversely, coadministration with strong CYP3A4 inducers resulted in a reduction in belumosudil exposure. However, coadministration with CYP3A4 inducers like efavirenz, nevirapine, and etravirine may reduce belumosudil exposure, potentially lowering its efficacy in immunosuppression. Regular monitoring is essential to evaluate GVHD status and optimize belumosudil treatment. 

In conclusion, managing ART during HSCT requires careful consideration of drug interactions and side effects to optimize treatment for hematologic cancer while maintaining effective HIV control. Consultation with an HIV specialist is highly recommended to navigate these complexities. 

**Table 1:** ART drug interactions and toxicities with lymphodepleting agents, immunosuppressive medications, and antifungal drugs.

Classes of ART	Drug Interactions	Toxicities	Lymphodepletion Interactions	Immunosuppression Interactions	Antifungal Interactions
NRTIs: (zidovudine (ZDV) [64], lamivudine (3TC) [65], emtricitabine (FTC) [66], abacavir sulfate (ABC) [67]	None	▪ All: lactic acidosis, hepatotoxicity ▪ ZDV: bone marrow suppression ▪ 3TC, emtricitabine: exacerbation of hepatitis B ▪ ABC: hypersensitivity reaction	None	None	None
NRTIs (TDF [68] and TAF [31])	▪ Both: coadministration of medications that reduce renal function or compete for active tubular secretion may increase tenofovir concentrations ▪ Both: substrate of P-glycoprotein (P-gp) and BCRP and medications that affect P-gp and BCRP metabolism may lead to changes in TAF absorption	▪ Both: exacerbation of hepatitis B, Lactic acidosis, ▪ TDF > TAF: Renal toxicity ▪ TDF > TAF: Decrease bone mineral density	None	▪ Cyclosporine, belumosudil: increase TDF/TAF exposure ▪ TDF/TAF and tacroliums: increase tacrolimus exposure, the possibility of nephrotoxicity	▪ isavuconazonium, posaconazole: may increase TDF/TAF exposure
First generation NNRTIs (efavirenz (EFV) [69], nevirapine (NVP) [70]	▪ Extensive metabolism by or inhibition of CYP450 system ▪ EFV, NVP: substrate and inducer of CYP3A4	▪ All: low barrier to resistance ▪ EFV, NVP: hepatotoxicity ▪ EFV: QT prolongation	▪ EFV, NVP: may decrease concentrations of cyclophosphamide	▪ EFV, NVP: may decrease concentrations of cyclosporine, tacrolimus, sirolimus, corticosteroids, ruxolitinib, and belumosudil	▪ EFV and posaconazole: may decrease exposure of posaconazole ▪ EFV and voriconazole: may decrease the exposure of voriconazole and increase the exposure of EFV ▪ NVP and voriconazole: may decrease the exposure of voriconazole and increase the exposure of NVP
Second generation NNRTIs (etravirine (ETR) [71], rilpivirine (RPV) [72], and doravirine [73])	▪ All: substrate of CYP34A ▪ ETR: inducer of CYP3A4 and inhibitor of P-gp	▪ All: effective against first-generation NNRTI resistance but still low barrier to resistance ▪ RPV: hepatotoxicity; supratherapeutic doses may prolong QT interval	▪ ETR: may decrease exposure to cyclophosphamide	▪ ETR: may decrease concentrations of tacrolimus, cyclosporine, sirolimus, corticosteroids, ruxolitinib, belumosudil	▪ ETR and voriconazole/posaconazole: may increase exposure of ETR ▪ RPV and voriconazole/posaconazole: may increase exposure of RPV
PIs, including boosting pharmacokinetic agents (darunavir (DRV) [74], cobicistat (COBI) [75], ritonavir (RTV) [76]	DRV: substrate of CYP3A4 COBI, RTV: substrate and inhibitor of CYP3A4	▪ RTV: hepatotoxicity, pancreatitis, PR interval prolongation, QT prolongation, hemophilia	▪ May increase concentrations of cyclophosphamide	▪ May increase exposure of cyclosporine, tacrolimus, sirolimus, corticosteroids, ruxolitinib, and belumosudil	▪ COBI/RTV and isavuconazonium; increase exposure of isavuconazonium; contraindicated ▪ COBI and voriconazole/posaconazole: may increase exposure of voriconazole/posaconazole and COBI ▪ RTV and voriconazole: decrease exposure of voriconazole; contraindicated ▪ RTV and posaconazole: both increase the risk of QT prolongation
INSTI (elvitegravir (EVG) [77] raltegravir (RAL) [78], dolutegravir (DTG) [79], bictegravir (BIC) [80], cabotegravir (CAB) [81]	▪ EVG, RAL, DTG, BIC: substrate of UGT1A1 ▪ EVG and BIC: substrate of CYP3A	▪ Generally well tolerated ▪ Raltegravir: hepato-toxicity	None	None	None
Entry inhibitors: enfuvirtide (T-20) [41], maraviroc (MVC) [44], fostemsavir (FTR) [45], ibalizumab-uiyk (IBA) [42]	▪ MVC: substrate of CYP3A, P-gp, OATP1B1, MRP2 ▪ FTR: substrate of CYP3A, P-gp, and BCRP	▪ T-20: injection site reaction, post-injection bleeding, hypersensitivity ▪ MVC: hepatotoxicity, cardiovascular events ▪ FTR: QT prolongation, elevations of hepatic transaminases in patients with HBV or HCV ▪ IBA: hypersensitivity, including infusion-related and anaphylactic reactions	None	▪ Cyclosporine: increase MVC, FTR, exposure	▪ MVC and posaconazole/voriconazole: may increase exposure of MVC; contraindicated in CrCL < 30 and iHD; dose reduction in MVC in CrCL > 30 ▪ FTR and posaconazole: may increase the exposure of FTR, increasing the risk of QT prolongation; contraindicated ▪ FTR and voriconazole both increase the risk of QT prolongation
Capsid inhibitor lenacapavir (LEN) [46]	Substrate of P-gp, UGT1A1. And CYP3A Moderate inhibitor of CYP3A, P-gp, and BCRP	▪ Injection site reactions	▪ May increase concentrations of cyclophosphamide	▪ May increase exposure of tacrolimus, cyclosporine, sirolimus, corticosteroids, ruxolitinib, belumosudil ▪ Cyclosporine: increase LEN exposure	NA

## 4. Prophylaxis and Prevention

Preventing infection is crucial for all populations, but for PWH, extra attention is essential to minimize morbidity and mortality. In the HIV/AIDS population, prophylaxis decisions are decided based on a patient’s CD4^+^ count or risk of infection recurrence. Prior to the availability of ART, opportunistic infections were highly prevalent due to immunosuppression. By the 1990s, guidelines were developed and recommended immunization and chemoprophylaxis for AIDS patients, which significantly improved morbidity and mortality. Current guidelines discuss preventing exposure to opportunistic pathogens, preventing disease, preventing disease recurrence, and initiation and discontinuation of prophylaxis [82]. The American Society of Clinical Oncology also has guidelines preventing infectious complications among HSCT recipients [83]. While there is some overlap between these guidelines, special care should be taken for PWH as certain treatments may further deplete cellular immunity and prolong lower CD4^+^ cell counts, positioning PWH at risk for opportunistic infections. Therefore, patients’ CD4^+^ cell counts should be closely monitored, and appropriate prophylaxis should be offered. Pre-HSCT screening for infection is essential to prevent post-HSCT infections and to determine appropriate prophylaxis and therapy according to published guidelines [83,84].

PWH face distinct risks of contracting infections, necessitating targeted prophylactic and treatment measures. These risks can arise from lifestyle choices, sexual activity, or differences in immune function. Strategies to prevent infections in PWH with significant immunosuppression focus on minimizing exposure whenever feasible. For instance, consistent condom use can help prevent sexually transmitted infections. To reduce the risk of gram-negative bacterial enteric infections like *Salmonella* spp., *Shigella* spp., and *Campylobacter* spp., to which PWH are particularly vulnerable to, it is essential to avoid consuming contaminated food or water. Thoroughly cooking meat, eggs, and seafood, consuming only pasteurized dairy products and juices, and exercising caution around bodies of water are also recommended. Maintaining rigorous hand hygiene, especially after contact with human or animal feces, is crucial. These measures also help mitigate the risk of other gastrointestinal opportunistic infections, such as Cystoisosporiasis, Cryptosporidiosis, and Microsporidiosis), to which PWH are particularly susceptible. For those who are not already seropositive, minimizing exposure to *Toxoplasma gondii* involves avoiding contact with cat feces. Preventing *Bartonella henselae* infections includes avoiding cat scratches and bites, practicing hand hygiene after contact with cats, and using flea control products on pets. *Bartonella quintana* infections can be reduced by avoiding exposure to human body lice, often found in crowded living conditions with inadequate hygiene. For PWH undergoing HSCT, understanding these infection risks is crucial, and these patients should receive counseling tailored to these risks and be encouraged to adopt a safe lifestyle accordingly [82]. 

Below, infections that warrant special attention for PWH will be highlighted, as they may have different risk factors compared to HSCT recipients. Table 2 includes infections that warrant prophylactic therapy for PWH. 

### 4.1. Mycobacterium Tuberculosis (MTB)

The United States Preventive Services Task Force (USPSTF) recommends screening for latent tuberculosis infection (LTBI) in populations at increased risk, including in PWH and those with other risk factors that elevate the likelihood of progression to active MTB disease, such as the use of immunosuppressive drugs [85]. In the general population without LTBI treatment, approximately 5 to 10% of infected persons will develop MTB disease, with about half of these cases occurring within the first two years of infection. The risk of developing MTB disease is significantly higher among immunocompromised hosts. Notably, PWH have a relative risk (RR) of 35 to 162, and those with hematologic malignancies have a RR of 8 to 72 [86]. For patients undergoing HSCT, the rate of MTB disease is 0.09% for autoHSCT and 1.05% for alloHSCT, usually manifesting over 100 days post-HSCT [87]. Identifying and treating LTBI is recommended as therapy decreases the risk of developing MTB disease by 60 to 90% [88,89].

### 4.2. *Pneumocystis Jiroveci* Pneumonia (PJP)

PWH whose CD4^+^ cell counts are below 200 cells/µL should continue PJP prophylaxis [82]. HSCT recipients are also at risk, and the introduction of prophylaxis has significantly reduced the incidence of PJP. However, cases still occur, particularly among those with acute or chronic GVHD who are receiving corticosteroids or other immunosuppressive medications. This is often due to non-compliance or inadequate prophylaxis [90]. Premature discontinuation of PJP prophylaxis has been associated with an increased incidence of PJP infections, underscoring the importance of maintaining long-term prophylaxis for individuals on immunosuppressive drugs and those with low CD4^+^ cell counts [83,91,92].

### 4.3. Endemic Fungal Infections

Endemic fungal infections such as *Histoplasma capsulatum*, *Coccidioides immitis,* and *Taloromyces (Penicillium) marneffei* are opportunistic in PWH. As these individuals are at heightened risk for these infections, prophylaxis is recommended for those with certain risk factors. For coccidioidomycosis, symptomatic infections are more likely in PWH with CD4^+^ counts below 250 cells/µL and those who are not virologically suppressed. In such cases, screening and prophylaxis are advised [82]. In endemic regions, histoplasmosis affects 2–25% of PWH and is often the first AIDS-defining infection in 50–75% of cases, with mortality rates ranging from 10 to 60%, depending on timely diagnosis and access to appropriate antifungal treatments beyond fluconazole [90]. *Talaromyces (Penicillium) marneffei* is an opportunistic infection in PWH for those residing in highly endemic regions in Southeast Asia or for those traveling to the region without access to ART. Primary prophylaxis is indicated for these individuals, and secondary prophylaxis should be maintained in those with CD4^+^ counts less than 100 cells/μL [82].

In contrast, these endemic fungal infections are rare among HSCT recipients, even in endemic regions. This rarity may be attributed to the widespread use of prolonged courses of mold-prophylactic azoles, which potentially suppress the emergence of these infections [90,93].

### 4.4. Cryptococcosis

*Cryptococcus neoformans* and *Cryptococcus gatti* are the primary pathogenic species responsible for the infection, and cryptococcal meningitis is a significant complication in PWH. Screening and prophylactic antifungal therapy are recommended to reduce the incidence of cryptococcal disease for individuals with positive serum *Cryptococcus* antigen (CrAg) test, and CD4^+^ counts less than 100 cells/µL [82]. In HSCT recipients, where antifungal prophylaxis is routine, these infections are rare, comprising less than 1% of all invasive fungal infections in this population [90]. For PWH undergoing HSCT, extended courses of antifungal prophylaxis may be necessary, especially in cases of prolonged low CD4^+^ counts.

### 4.5. Toxoplasma Gondii Encephalitis (TE)

For PWH, primary prophylaxis for Toxoplasma IgG-positive patients should initiate at a CD4+ count of less than 100 cells/μL and could be discontinued once the CD4^+^ count has increased to over 200 cells/μL for at least three months. All regimens that are recommended for TE prophylaxis are also effective as PJP prophylaxis. Secondary prophylaxis should be maintained until CD4^+^ cell counts exceed 200 cells/μL for at least six months and restarted once CD4+ cell count is below 200 cells/μL [82].

Among HSCT recipients, toxoplasmosis is an uncommon but serious infection that usually occurs due to reactivation in those with GVHD or in umbilical cord HSCT recipients. Although rare, donor-derived infections have also been reported. For autoHSCT recipients, the risk is low unless CD34 selection is performed, or additional T cell immunosuppressive therapies are administered. For seropositive alloHSCT recipients, recent European guidelines recommend the use of quantitative Toxoplasma PCR testing to identify infection pre-emptively, even in those on trimethoprim-sulfamethoxazole (TMP-SMX) prophylaxis, as this occasionally fails. Prophylaxis should continue throughout the period of immunosuppression and CD4^+^ lymphopenia [94], particularly PWH who may be at a heightened risk. 

### 4.6. Cystoisosporiasis

Prophylaxis with TMP-SMX, which is also used for PJP and TE, is associated with a lower incidence of infection in PWH. Secondary prophylaxis is recommended until CD4^+^ counts are sustained above 200 cells/μL for at least six months and should be initiated when CD4+ count is below 200 cells/μL. Among HSCT recipients, there is a risk of developing disseminated infections, particularly in those with lower CD4^+^ counts during immunosuppression for the treatment of GVHD [95,96,97,98]. Recurrences have been reported with ongoing immunosuppression [99]. For PWH undergoing HSCT, monitoring of CD4^+^ counts should be followed before discontinuing of prophylaxis to ensure continued protection.

### 4.7. Mycobacteria Avium Complex (MAC)

MAC is ubiquitous in the environment and poses a risk of disseminated infection in PWH with CD4^+^ cell counts below 50 cells/μL. Although the risk has diminished with ART, factors such as elevated HIV RNA levels indicating ongoing viral replication despite ART, prior or concurrent opportunistic infections, and underlying T-cell dysfunction are still associated with increased susceptibility [100]. Primary prophylaxis is no longer recommended for PWH who promptly initiate ART. However, it remains advisable for those who remain viremic or have limited options for a fully suppressive ART regimen [82].

Non-tuberculous Mycobacterial (NTM) infections have been documented among HSCT recipients, manifesting as cutaneous, pulmonary, or catheter-associated infections [101]. The incidence of pulmonary NTM infections post-HSCT surpasses that in the general population [102]. For instance, a study at one institution found that 2.7% of alloHSCT recipients developed NTM disease, primarily pulmonary (93.3%), but disseminated (6.7%) cases occur. Risk factors included severe chronic GVHD and CMV viremia [103]. In HSCT recipients, skin and lung lesions should undergo biopsy and acid-fast bacilli cultures. Mycobacterial infections associated with catheters can be diagnosed via tunnel or blood cultures. While data are limited, HSCT can be successful in patients with adequately treated NTM infection [104]. Given the prolonged CD4^+^ lymphopenia risk among PWH undergoing HSCT, primary prophylaxis remains indicated for those with persistently low CD4^+^ cell counts to prevent MAC.

### 4.8. Cytomegalovirus (CMV)

CMV is a common virus that generally causes mild or asymptomatic infections in healthy individuals. However, for PWH who are immunosuppressed, particularly those with, but for PWH who immunosuppressed, with CD4^+^ cell counts below 50 cells/μL, there is a risk of developing CMV end-organ disease. This condition is characterized by the involvement of specific organs, with clinical manifestations varying depending upon the affected system. The most common presentations of CMV end-organ disease include retinitis, followed by colitis, esophagitis, and neurologic involvement. Unlike in HSCT recipients, pneumonitis is uncommon in this population. Diagnosis in PWH relies heavily on clinical findings with tissue biopsy for definitive confirmation when possible. Blood tests to detect CMV antigen, culture, or PCR are not recommended for diagnosing end-organ disease because of their poor positive predictive value in PWH. A negative serum or plasma PCR does not exclude CMV end-organ disease. Furthermore, CMV viremia may be present in the absence of end-organ disease in PWH with low CD4^+^ counts. Additionally, positive CMV cultures from the colon, esophagus, or lung brushings do not confirm clinical disease without corresponding histopathological changes, as shedding can occur in the absence of clinical disease. Primary prophylaxis is not routinely recommended for PWH at risk (CD4^+^ < 100 cells/μL and CMV viremia) for end-organ disease. Instead, early recognition of symptoms and prompt initiation of therapy in at-risk individuals are crucial. Ophthalmologic examination every three to four months should be considered in PWH with CD4^+^ counts below 50 cells/μL [82].

In contrast, CMV serostatus significantly impacts outcomes following alloHSCT, where CMV-positive serology correlates with increased morbidity and mortality due to its immunomodulatory effects. A pre-emptive approach involves weekly screening with CMV PCR and initiation of antiviral therapy upon detecting viremia above a defined threshold, preventing progression to end-organ disease. Since 2017, many centers have adopted primary prophylaxis with letermovir, which may also serve as secondary prophylaxis in high-risk patients [105]. As PWH may have an increased risk of developing CMV reactivation, including auto-HSCT recipients due to prolonged CD4^+^ lymphopenia [4,25] prophylactic strategies specific to this population remain understudied. Letermovir, although lacking penetration into the central nervous system or eyes, has been reported to effectively treat CMV retinitis in non-HIV patients [106]. At our institution, we have successfully employed letermovir as secondary prophylaxis in one autoHSCT and one alloHSCT in patients with pre-existing CMV retinitis, highlighting the need for further research in this area.

### 4.9. Bartonella spp.

PWH are at increased risk for *Bartonella* spp. infections, which can cause bacillary angiomatosis, peliosis hepatis, bacteremia, osteomyelitis, central nervous system infections, and infective endocarditis, especially in those with CD4^+^ cell counts below 50 cells/μL. While primary chemoprophylaxis is not recommended, macrolide use has been shown to be protective against *Bartonella* infections [82]. Although *Bartonella* infections have rarely been described among HSCT recipients, they should be considered in those with fever of unknown origin in this population [107,108].

### 4.10. Mold Infections

Invasive mold infections are uncommon in PWH, with the estimated incidence of aspergillosis among AIDS patients reported as 3.5 cases per one thousand person-years in national databases and slightly higher in hospitalized individuals at 0.43%. Risk factors include neutropenia or corticosteroid use, concomitant PJP infection, and CD4^+^ counts below 50–100 cells/µL. A recent meta-analysis spanning from 1985 to 2021 highlighted an elevated mortality rate of 83% in PWH with *Aspergillus* spp. infections, which decreased to 31% in the post-ART era with combined ART and antifungal treatment [109]. 

Conversely, HSCT poses a significantly higher risk for invasive fungal infections (IFI), particularly alloHSCT, primarily due to prolonged neutropenia, GVHD, CMV reactivation, high-dose steroid use, transplant-associated microangiopathy, and underlying acute myelogenous leukemia (AML) or myelodysplastic syndrome (MDS). Mortality rates can reach up to 50%. Routine use of antifungal prophylaxis in HSCT has reduced infection rates but altered the epidemiology of IFIs among recipients [110]. HSCT guidelines recommend mold-active prophylaxis when the risk of invasive aspergillosis exceeds 6%, particularly in patients with AML or MDS or during GVHD treatment [83]. PWH undergoing HSCT and requiring prolonged immunosuppression with low CD4^+^ counts may necessitate extended courses of prophylaxis to effectively mitigate IFI risks.

### 4.11. Clostridioides Difficile Infection (CDI)

CDI is prevalent among PWH, particularly those with a low CD4^+^ count of less than 50 cells/μL, which is an independent risk factor. However, antimicrobial prophylaxis specifically targeting CDI is not routinely recommended for PWH alone. HSCT recipients also experience an approximately nine-times higher incidence of CDI compared to hospitalized patients overall [111]. CDI in this population is associated with prolonged hospital stays, increased morbidity and mortality. Moreover, the risk of one or more CDI recurrences among HSCT recipients has been reported to be as high as 20%, attributable to ongoing risk factors such as chemotherapy, broad-spectrum antibiotic use, and GVHD [112]. While current guidelines do not advocate for prophylactic measures for individuals at high risk for CDI [113], secondary prophylaxis with oral vancomycin may be considered in the HSCT population, as this approach has demonstrated effectiveness in reducing the rate of CDI in this population [112].

### 4.12. Human Papilloma Virus (HPV)

Cervical cancer screening is recommended for women with HIV upon diagnosis and subsequently at varying intervals based on age and risk factors and due to HPV co-infection. This screening is especially crucial for those who undergo HSCT, as there is evidence suggesting an increased risk of cervical cancer post-HSCT, particularly in individuals with concurrent HPV infection. Therefore, it is advised that women HSCT recipients follow cervical cancer screening guidelines recommended for women with HIV [114]. 

HPV infection is also associated with anal cancer in PWH, affecting both men and women, as well as other immunocompromised hosts like solid organ transplant recipients [115]. Screening for anal disease is recommended via digital anorectal examination for all PWH. Additionally, for men who have sex with men (MSM), transgender women, or transgender men, anal pap testing is recommended to detect potentially cancerous cytologic abnormalities [116]. 

### 4.13. Sexually Transmitted Infections (STIs)

For PWH, routine screening for STIs is essential. This includes testing for syphilis, gonorrhea, chlamydia, and trichomoniasis (for those having receptive vaginal sex). Additional testing for gonorrhea and chlamydia in the pharynx and rectum may be considered for both males and females, depending on their sexual behaviors and potential exposure. The frequency of screening depends on individual risk behaviors and local epidemiology [117]. Rectal examinations and swabs should be tailored to individual risk behaviors and local epidemiologic factors. During periods of neutropenia, it is recommended to avoid rectal swabs to minimize the risk of infection due to compromised skin and mucosal integrity [118]. If STIs are detected, prompt treatment and counseling on safe sex practices should be provided. 

**Table 2:** Opportunistic infections in PWH undergoing HSCT that require antimicrobial prophylaxis.

Disease and HIV CD4^+^ Cell Count (Cells/μL) Risks	Treatment and Primary Prophylaxis for PWH	Secondary Prophylaxis for PWH	Special Considerations for HSCT Recipients
*Mycobacterium tuberculosis* (TB) All CD4^+^ counts	▪ Positive screening test for latent TB (LTBI), no evidence of active MTB, and no prior treatment for active MTB or LTBI or ▪ Close contact with a person with infectious MTB, with no evidence of active MTB, regardless of screening test results.	Not applicable	The same recommendations in HSCT recipients.
*Pneumocystis jirovecii* pneumonia (PJP) CD4^+^ cell count ≤ 200 cells/μL or CD4^+^ percentage of <14% lymphocyte count	▪ Primary prophylaxis until CD4^+^ cell count is >200 cells/μL for >3 months.	▪ If PJP occurs at a CD4^+^ count >200 cells/μL while not on ART, discontinuation of prophylaxis can be considered once HIV RNA levels are undetectable ≥ 3–6 months. ▪ If PJP occurs at CD4^+^ count > 200 cells/μL while on ART, continue prophylaxis for life, regardless of CD4^+^ cell count.	▪ In autoHSCT, prophylaxis is recommended in those receiving myeloablative conditioning regimens, graft manipulations (e.g., CD34 selection), those receiving high-dose glucocorticoids, and those who received purine analog. Prophylaxis is 3–6 months post-autoHSCT or longer for those requiring further immunosuppressives. ▪ In alloHSCT recipients, PJP prophylaxis starts after neutrophil engraftment and continues for 6 months, or longer in those on immunosuppression. ▪ For PWH, monitoring of CD4^+^ counts should be followed before discontinuing prophylaxis.
Coccidioidomycosis CD4^+^ cell count ≤ 250 cells/μL	▪ Screen if previously traveled or lived in endemic areas. ▪ Those with new positive IgM or IgG serologic test should start primary prophylaxis until CD4^+^ count ≥ 250 cells/μL with virologic suppression on ART.	▪ May consider stopping secondary prophylaxis in those with history of pneumonia and clinically respond to ≥12 months antifungal therapy with CD4^+^ count ≥ 250 cells/μL and receiving effective ART, with monitoring for recurrence with serial chest imaging and Coccidioides serology. ▪ For people with diffuse pulmonary, disseminated, or meningeal disease, suppressive therapy should be continued indefinitely.	▪ No guidelines for HSCT but consider pre-HSCT. ▪ Primary prophylaxis in those who visit or live in Coccidioides-endemic areas for first 100 days post-HSCT, and longer if remains immunosuppressed. ▪ Secondary prophylaxis should continue while patients remain immunosuppressed [119].
*Histoplasma capsulatum* infection CD4^+^ cell count ≤ 150 cells/μL	▪ Prophylaxis for those at risk from occupational exposure or living in a region with a hyperendemic (>10 cases/100 patient years). ▪ May discontinue primary prophylaxis after CD4^+^ count > 150 cells/μL and undetectable HIV viral loads for 6 months.	▪ May discontinue secondary prophylaxis if the patient received therapy for >1 year and has negative fungal cultures and negative serum or urine Histoplasma antigen and undetectable viral loads and CD4^+^ count ≥ 150 cells/μL for ≥6 months on ART	▪ Rare but associated with a high mortality among HSCT recipients [120,121] ▪ For PWH, monitoring of CD4^+^ counts should be followed before discontinuing prophylaxis.
*Talaromyces (Penicillium) marneffei* CD4^+^ T cell count < 100 cells/μL	▪ Primary prophylaxis is indicated for those who reside in highly endemic regions in the Southeast *or* who are from countries outside of the endemic region, and travel to the region and do not have access to effective ART	▪ Secondary prophylaxis with chronic maintenance therapy should be performed until CD4^+^ count > 100 cells/μL for ≥6 months on ART and HIV viral load is suppressed for >6 months.	▪ Reported in HSCT recipients. ▪ For PWH, monitoring of CD4^+^ counts should be followed before discontinuing prophylaxis.
Cryptococcosis	▪ Routine surveillance testing for serum CrAg is recommended for patients with CD4+ counts ≤ 100 cells/µL. A positive test should prompt evaluation for central nervous system infection. ▪ Prophylactic antifungals can reduce the frequency of primary disease, and in the United States, prophylaxis is recommended for those with a positive serum CrAg test.	▪ Discontinue secondary prophylaxis if the patient completed initial (induction and consolidation) therapy, received at least 1 year of antifungal therapy, and remains asymptomatic of cryptococcal infection, and CD4^+^ count ≥ 100 cells/μL with suppressed plasma HIV RNA in response to ART.	▪ Rare among HSCT recipients [90]. ▪ For PWH, monitoring of CD4^+^ counts should be followed before discontinuation of prophylaxis.
*Cystoisosporiasis* CD4^+^ cell count ≤ 200 cells/μL	▪ Prophylaxis with trimethoprim-sulfamethoxazole (TMP-SMX) (also used for PJP, TE) is associated with a lower incidence of infection.	▪ Secondary prophylaxis with TMP-SMX while CD4^+^ counts are <200 cells/μL.	▪ In HSCT can develop disseminated infection, which usually occurs in patients with lower CD4+ counts during immunosuppression for treatment of GVHD [95,96,97,98]. ▪ Recurrences may occur with immunosuppression [99]. ▪ For PWH, monitoring of CD4+ counts should be followed before discontinuing prophylaxis
*Toxoplasma gondii* encephalitis (TE) CD4^+^ T cell count < 100 cells/μL	▪ Primary prophylaxis in Toxoplasma IgG-positive patients until CD4+ count is >200 cells/μL in response to ART. ▪ All regimens recommended for primary prophylaxis against toxoplasmosis are also effective as PJP prophylaxis.	▪ After completion of acute therapy, patients should remain on chronic maintenance therapy and remain free of signs/symptoms of TE and CD4 count > 200 cells/μL for >6 months.	▪ Uncommon in HSCT recipients. ▪ For those seropositive, European guidelines recommend screening by quantitative PCR to identify infection as a pre-emptive strategy [94].
*Cystoisosporiasis* CD4^+^ cell count ≤ 200 cells/μL	▪ Prophylaxis with TMP-SMX (also used for PJP, TE) is associated with a lower incidence of infection.	▪ Secondary prophylaxis with TMP-SMX while CD4^+^ counts < 200 cells/μL.	▪ In HSCT can develop disseminated infection during immunosuppression, and recurrences can occur [95,96,97,98,99]. ▪ For PWH, monitoring of CD4+ counts should be followed before discontinuing prophylaxis
*Mycobacterium avium* complex (MAC) disease CD4^+^ T cell count < 50 cells/μL	▪ Primary prophylaxis is not recommended for those who immediately initiate ART ▪ Primary prophylaxis is recommended for those not on fully suppressive ART after ruling out active disease.	▪ Secondary prophylaxis may be discontinued if the patient has completed ≥12 months of therapy and no signs/symptoms of MAC infection and CD4^+^ cell counts > 6 months > 100 cells/μL on ART.	▪ MAC infections have been documented among HSCT recipients, manifesting as cutaneous, pulmonary, or catheter-associated infections [101] ▪ For those with prolonged CD4^+^ lymphopenia who will be at risk of developing MAC, primary prophylaxis remains indicated.
Cytomegalovirus CD4^+^ T cell count < 50 cells/μL	▪ Primary prophylaxis is not recommended in PWH	▪ Consider discontinuation of secondary prophylaxis after CMV treatment for 3–6 months and CD4 count > 100 cells/μL for >3–6 months. Therapy should be discontinued only after consultation with an ophthalmologist, and routine ophthalmologic follow-up is recommended after stopping therapy. ▪ Restarting secondary prophylaxis when CD4^+^ T cell count is <100 cells/μL.	▪ In HSCT, primary prophylaxis with letermovir, which may also serve as secondary prophylaxis in high-risk patients [105] ▪ As PWH undergoing HSCT may have an increased risk of developing CMV reactivation, primary prophylaxis may be indicated for longer periods with prolonged immunosuppression or CD4^+^ lymphopenia.

## 5. Vaccinations

PWH are at higher risk for contracting certain vaccine-preventable diseases due to immune function, lifestyle choices, and sexual activity. It is recommended that PWH remain up-to-date with vaccinations against hepatitis A and B, meningococcus, pneumococcus, influenza, SARS-CoV-2, and tetanus, diphtheria, and pertussis, irrespective of their CD4^+^ cell count [82]. 

After HSCT, recipients may lose immunity to various pathogens and consider these individuals as “never vaccinated”, necessitating a complete vaccination schedule based on their age, geography, and local epidemiology. Guidelines advise initiating vaccinations 3–6 months post-HSCT, although some centers may delay certain vaccines until 12 months post-HSCT due to factors like delayed reconstitution or specific immunomodulating therapies. However, there are no data that support waiting to start vaccination based on a specific lymphocyte panel, and delaying vaccination increases the period during which patients are vulnerable to vaccine-preventable illnesses. Inactivated vaccines are safe post-HSCT, although they may initially elicit a reduced immune response compared to healthy individuals. This response typically improves over 2–3 years post-HSCT. Live-attenuated vaccines are recommended cautiously, typically at least 24 months post-HSCT, to mitigate the risk of vaccine-transmitted disease. Vaccinating close contacts, including live-attenuated vaccines such as MMR and VZV, if applicable, is encouraged to enhance protection for HSCT recipients. Intranasal live attenuated influenza vaccine or polio vaccine should be avoided due to prolonged viral shedding and transmission risks [122].

For PWH who undergo HSCT, guidelines for HSCT recipients apply, with special attention to meningococcal disease, where PHW are at a 5 to 24 increased risk compared to those without HIV. Urban outbreaks have been noted among MSM, including those with HIV. Guidelines strongly advocate meningococcal vaccination (groups A, C, w, and Y) for all adult PWH, irrespective of age [82].

Additionally, the Advisory Committee on Immunization Practices recommends MPOX vaccination for individuals at risk due to occupational exposure or specific risk factors, including sexual behaviors such as multiple partners or engagement in high-risk sexual activities in areas with known MPOX transmission [123].

## 6. Cure

There are five known cases of a cure for HIV-1 following HSCT from donors homozygous for a thirty-two base pair deletion in the CCR5 gene (CCR5Δ32/Δ32). This mutation confers resistance to R5-tropic HIV-1 by disabling the CCR5 receptor on CD4^+^ cells [124]. Each of these cases varied in the duration of HIV-1 infection prior to HSCT, the type of lymphodepletion regimen used (both myeloablative and non-myeloablative), and the source of the stem cells. The successful cure appears contingent upon infection with R5 tropic virus and achieving 100% chimerism of the graft [22].

As the HIV population ages and the risk of hematologic malignancies rises, HSCT will become increasingly more prevalent among PWH with poor prognostic malignancies. With the availability of potential donors possessing permissive genetics that allow for the selection of CCR5Δ32/Δ32 and HLA matching requirements are modified with post-HSCT cyclophosphamide, there is optimism for more potential cures among PWH and hematologic malignancies. Research opportunities and trials in this area show promise and merit promotion and support.

## 7. Conclusions

In conclusion, the integration of HSCT into the treatment paradigm for hematologic malignancies in PWH reflects considerable progress, with outcomes comparable to the general population. However, the complexity of managing polypharmacy and potential drug interactions underscores the need for a personalized approach that addresses ART selection, infection risk assessment, and comprehensive supportive care measures. By navigating these challenges thoughtfully, healthcare providers can optimize outcomes and improve the quality of care for this unique patient population undergoing HSCT.
